# Prevalence and factors associated with smartphone addiction among medical students at King Abdulaziz University, Jeddah

**DOI:** 10.12669/pjms.344.15294

**Published:** 2018

**Authors:** Alaa Aziz Alhazmi, Sami H. Alzahrani, Mukhtiar Baig, Emad M. Salawati, Ahmad alkatheri

**Affiliations:** 1 Alaa Aziz Alhazmi, Public Health Administration, Ministry of Health, Makkah, Saudi Arabia; 2 Sami H. Alzahrani, Family and Community Medicine Department, Faculty of Medicine, King Abdulaziz University, Jeddah, Saudi Arabia; 3 Mukhtiar Baig, Clinical Biochemistry/Medical Education, Faculty of Medicine, Rabigh, King Abdulaziz University, Jeddah, Saudi Arabia; 4 Emad M. Salawati, Family and Community Medicine Department, Faculty of Medicine, King Abdulaziz University, Jeddah, Saudi Arabia; 5 Ahmad alkatheri, Family and Community Medicine Department, Faculty of Medicine, King Abdulaziz University, Jeddah, Saudi Arabia

**Keywords:** Medical students, Obesity, Saudi Arabia, Smartphone addiction

## Abstract

**Objective:**

To investigate smartphone addiction among medical students and to determine factors associated with smartphone addiction among sixth-year medical students at King Abdulaziz University, Jeddah.

**Methods:**

This cross-sectional study was conducted on 203 sixth-year medical students at the Faculty of Medicine, King Abdulaziz University, Jeddah, Saudi Arabia, during July 2017. Data analysis was done using SPSS-20.

**Results:**

The number of completed questionnaires received was181 out of 203, making a response rate of 89%. There were 87 male respondents (48.1%) and 94 female respondents (51.9%). The overall prevalence of smartphone addiction was 66 (36.5%). There is a statistically significant relationship between daily hours of smartphone usage and smartphone addiction (p<0.02). Out of 66 addicted students, 24 (55.8%) students reported using their smartphone more than five hours daily, 17(34.7%) students were using it 4 to 5 hours daily, 13 (27.7%) students were using it 2 to 3 hours daily and 12(28.6%) students were using it less than two hours daily. The study showed no statistically significant relationship between smartphone addiction and smoking statusor degree of obesity. There was a significant association between the total score on the smartphone addiction scale and daily usage hours (p-value<0.005).

**Conclusion:**

The overall prevalence of smartphone addiction was high among our study participants. The smartphone addiction was associated with daily hours of smartphone usage.

## INTRODUCTION

The development of smartphones has caused a dramatic change in societies worldwide, and it has transformed communication among people of all ages. It is one of the best tools that have integrated “communication, education, and entertainment.” 1 It seems that the famous idiom “to have the world in the palm of your hand” is the best characterization of smartphones. According to a recent report, the Kingdom of Saudi Arabia ranked third in the world in terms of population using smartphones, at 72.8%.^1^

Smartphones have become so common that the number of mobile cellular subscriptions is expected to reach almost 6 billion worldwide by the end of 2020.^2^ A study at King Abdulaziz University (KAU) in Jeddah pointed out that almost all of the medical students there have smart devices and students (89.1%) had installed different medical apps on their smart devices.^3^ A recent study reported that 90% of the KAU students texted while driving and overall use of mobile phone for calling and texting was high.^4^

Modern phones with high-resolution touch screens serve as portable media players, digital cameras, video cameras, navigation units, and internet browsers. Further, they are used for social networking, checking and sending emails, appointment scheduling, shopping, gaming, and entertainment. These advantages have made them an integral part of human life.On the other hand, unfavorable results of smartphone use that have been reported in the literature include compulsively checking the device^5^, uncontrollable daily usage, and unwanted distress.^6^ In the end, smartphones can be addictive.^7^ One study showed that, without their mobile phone, many people feel incomplete and worried.^8^

Smartphones have become part and parcel of our daily lives; these devices are not merely mobile phones but are in fact handheld computers keeping us on schedule in our day-to-day activities and allowing us to keep in touch with friends and family. In addition to the uses already listed, smartphones are used for taking part in video conferences, downloading favorite songs and movies, and a host of other functions that were once thought impossible to perform on such a compact device.^9^

“Addiction is considered by WHO as dependence, as the continuous use of something for the sake of relief or stimulation, which often causes cravings when it is absent.”^10^ The excessive use of smartphones to a level where it interferes with the daily lives of users is thus considered to be smartphone addiction.^11^

Smartphone addiction is a worldwide phenomenon in a range of 9.3% to 48% of the population.^12^-^14^ A study in Saudi Arabia reported that smartphone addiction is associated with negative impacts on levels of energy, sleep, eating behaviors, body weight, exercise, and academic achievements.^15^

There are some studies on this topic focused on Saudi Arabia;^14^,^15^ however, in our university, there is a scarcity of data. Therefore, the present study was designed to investigate smartphone addiction among medical students and to determine factors associated with smartphone addiction among sixth-year medical students at KAU.

## METHODS

This cross-sectional, questionnaire-based study was conducted on 203 sixth-year medical students at the Faculty of Medicine, KAU, in Jeddah, Saudi Arabia, during July 2017. The sample size was calculated by using the RAOSOFT sample size calculator, assuming a 95% confidence level, 5% sampling error, and 50% probability of occurrence. The minimum calculated sample was 177. Assuming a non-response rate of 15%, the sample size was increased to 203. The sample was proportionally stratified according to gender, and then the required participants were selected randomly from the list of student names.

The data collected with regard to personal characteristics included age, sex, marital status, hours of daily smartphone usage, smoking status, height, and weight.

We used the already published smartphone addiction scale (SAS) short version questionnaire, and the internal consistency and concurrent validity of SAS were verified (Cronbach’s alpha=0.967).^16^

The SAS questionnaire contains 10 questions with response choices from 1 to 6, where 1 is strongly disagree and 6is strongly agree. Totals of responses were calculated and compared to cutoff points of 31 for males and 33 for females.Students who scored higher than the cutoff were considered to be addicted. All sixth-year medical students were included. A pilot study had been conducted with 20 sixth-year medical students who were excluded from the main study.

The researcher (with the help of female professional data collectors) distributed the questionnaire to students after they had finished their academic sessions. The questionnaires were collected on the same day. The data was verified by hand, then coded and entered into a personal computer. The study was approved by the Research Ethics Committee of the Faculty of Medicine, KAU, Jeddah, and consent forms weresigned by all participants.

### Statistical analysis

Data analysis was done by using Statistical Package for the Social Sciences Version 20 (SPSS, 20) software. The Chi-square test was used to compare qualitative variables. The Kolmogorov–Smirnov (K-S)test was conducted to assess the normality of the data and showed that the data in this study were not normally distributed. Thus, nonparametric tests, including the Mann–Whitney U test and the Kruskal–Wallis H test, were used for continuous variables. The p-value of less than 0.05 was used as the significant level in all the analyses.

## RESULTS

The number of completed questionnaires received was 181 out of 203, making the response rate 89%. The mean age of students in this study was 24 years. The number of male respondents was 87 (48.1%), females were 94 (51.9%), married respondents were 19 (10.5%), and single respondents were 162 (89.5%). According to the body mass index scale, 83 respondents (45.9%) were overweight, 44 (24.3%) were obese, 43 (23.8%) had normal weight, and 11 (6.1%) were underweight (Table-I). The overall prevalence of smartphone addiction was 66 (36.5%), and males 38 (57.6%) reported more smartphone addiction than females 28 (42.4%); however, the difference was not significant.

**Table-I:** Demographic variables of the study participants.

Variables	No. (%)
Age (Mean ± SD)	24.09 ±0.443
***Gender***	
Male	87(48.1)
Female	94(51.9)
***Marital status***	
Married	19(10.5)
Single	162(89.5)
***Daily hours of smartphone usage***	
Less than 2 hours	42(23.2)
2–3 hours	49(26.0)
4–5 hours	47(27.1)
More than 5 hours	43(23.8)
***Smoking status***	
Never	91(50.3)
Former	51(28.2)
Current	39(21.5)
***Body mass index interpretation***	
Underweight	11(6.1)
Healthy weight	43(23.8)
Overweight	83(45.9)
Obese	44(24.3)

**Fig.1:**
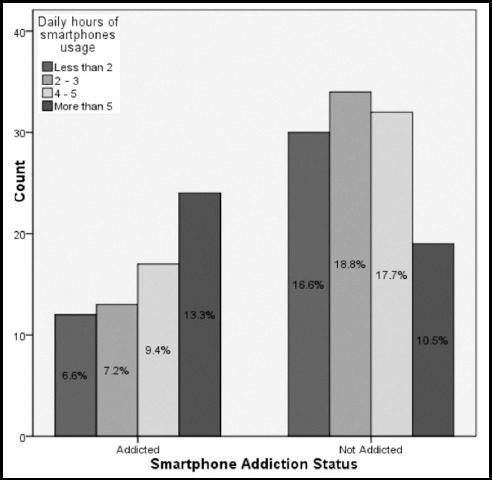
Daily hours of smartphone usage among participants.

There is a statistically significant relationship between daily hours of smartphone usage and smartphone addiction (p<0.02). Out of 66 addicted students, 24(55.8%) students reported using a smartphone for more than 5 hours daily, 17 (34.7%) students used a smartphone 4–5 hours daily, 13(27.7%) used a smartphone 2–3 hours daily, and 12 (28.6%) students used a smartphone less than 2 hours daily (Fig.1, Table-II). The study showed no statistically significant relationship between the smoking status and degree of obesity of the students and smartphone addiction (Table II and III).

**Table-II:** Factors associated with smartphone addiction.

Factors	Smartphone Addiction Status				p value

	Not addict		Addict		

	No.	%	No.	%	
Gender					*NS
Male	49	56.3	38	43.7	
Female	66	70.2	28	29.8	
Daily hours of smartphone usage					0.021
Less than 2	30	71.4	12	28.6	
2–3	34	72.3	13	27.7	
4–5	32	65.3	17	34.7	
More than 5	19	44.2	24	55.8	
Smoking status					*NS
Never	55	60.4	36	39.6	
Former	35	68.6	16	31.4	
Current	25	64.1	14	35.9	
Body mass index interpretation					*NS
Underweight	7	63.6	4	36.4	
Healthy weight	28	65.1	15	34.9	
Overweight	54	65.1	29	34.9	
Obese	26	59.1	18	40.9	

* =not significant

**Table-III:** Factors associated with smartphone addiction.

Variables	(%) No.	Mean rank	p-value
***Gender***			NS
Male	87	93	
Female	94	89	
***Daily hours of smartphone usage***			0.005
Less than 2 hours	42	80	
2–3 hours	47	77	
4–5 hours	49	93	
More than 5 hours	43	113	
***Smoking status***			NS
Never	91	92	
Former	51	89	
Current	39	89	
***Body mass index interpretation***			NS
Underweight	11	85	
Healthy weight	43	91	
Overweight	83	87	
Obese	44	98	

Out of 66 addicted students, the mean rank of the total score on the SAS was higher among males (93) than among females (89) and it was also higher for those students whose daily smartphone totaled more than 5 hours (113). There was a significant statistical association between the total score on the SAS and daily usage hours (p- value <0.005) (Table-III).

## DISCUSSION

Our study results showed that out of 181 participants, the overall prevalence of smartphone addiction was 36.5%, which is very high. One of the reasons for this high prevalence could be that a lot of educational material is available on the internet, and students feel more comfortable using a smartphone than a laptop or desktop computer. Another reason could be that KAU is using course management software (Blackboard, Bb), and most module coordinators use this software for uploading educational material like PowerPoint slides, assignments, and study guides. Bb is also used for formative assessments. Therefore, the use of a smartphone for educational purposes could be one of the important contributing factors.

Several studies have reported variable results regarding smartphone addiction in the populations of Switzerland (16.9%),^17^ Tunis (31.7%),^18^ Korea (16%),^19^ India (37%),^8^ Iran (9.3%),^12^ and Belarus(10%).^20^ The difference in our results when compared to other studies could be due to different sample sizes, different population characteristics, or use of different tools for assessing levels of addiction

Our study participants showed a statistically significant relationship between daily hours of smartphone usage and smartphone addiction (p<0.02). More than half of the addicted students were using their smartphone more than five hours daily, and one-third of the students were using a smartphone 4 to 5 hours daily. There was a significant statistical association between the total score on the SAS and daily usage hours (p-value <0.005).

Our results are similar to other studies.^17^,^21^ It seems that positive socioeconomic conditions mean that a significant portion of the population owns smart devices and luxury vehicles. However, the use of a smartphone for long hours is not good for medical students due to the fact that medical studies require more time and concentration for studying and understanding concepts. Therefore, students and other concerned bodies should think about this serious issue and take steps to reduce the prevalence of this addiction

Our study showed that addiction is higher among males than females, which is similar to a few other studies,^8^,^14^ while Alosaimi FD et al. (2016) and Matar Boumosleh J and Jaalouk D (2017) reported that gender was not associated with smartphone addiction in their studies.^15^,^22^ A Turkish study reported that among university students, smartphone addiction was more prevalent in those who first used mobile phones at a younger age, those with a Type-A personality, those with a low financial status, and those who used a smartphone for more than five hours a day. They also reported more sleep disturbancesassociated with high levels of smartphone addiction.^21^ The relationship between mobile phone use at a young age and smartphone addiction was also reported by another study that additionally showed anxiety and depression to be predictors of smartphone addiction.^22^

Our findings could be explained by the fact that in Saudi Arabia, there are fewer social activities and entertainment opportunities, and it is therefore likely that students opt to remain at home and use smartphones for social and entertainment activities.

The present study showed no statistically significant relationship between smartphone addiction and either smoking status or degree of obesity. Similar to our results, Haug S et al. (2015) reported that among students in Switzerland, the use of alcohol and tobacco were not related to smartphone addiction.^17^ Few studies highlighted smartphone-related symptoms like sleep disturbances, concentration impairment, hearing problems, facial dermatitis, and headaches among medical students.^18^

A recent study by Tamura H et al. (2017) reported that Japanese adolescents who used a mobile phone for 5 hours or more per day suffered from insomnia. Additionally, study participants who had excessive use of a mobile phone for social networking and online chats exhibited higher levels of depression than participants using the phones for playing games, viewing videos, or searching the internet.^23^

In the present study, 70% of participants were either overweight or obese. This high rate of obesity is similar to a recent report published by KAU.^24^ Another report has suggested that smartphone addiction is more prevalent in students having a lack of physical activity and higher stress levels.^17^ It is most likely that our study participants have physical inactivity because of obesity and that they have stress because of educational pressure and obesity. Thus, both could be contributing factors to increased mobile addiction among medical students.

Our results suggest that excessive use of the smartphone is associated with mobile phone addiction and that this addiction could potentially pose a health risk. Therefore, it is recommended that excessive use should be avoided, and in this regard, awareness campaigns should be started by both governmental and non-governmental organizations. The mass media could play an important role in increasing general awareness among the public with regard to the dangerous impacts smartphone device use has on human health and behavior and the ultimate consequences to society.

### Limitations of the study

Our study has some limitations, including a small sample size and the fact thatthe sample group was collected from one class and from one medical college. Therefore, the results of the present study cannot be generalized. It is possible that the prevalence of smart phone addiction is greater or lesser among students at other universities in Saudi Arabia.

## CONCLUSION

The overall prevalence of smartphone addiction was high among our study participants, and this smartphone addiction was associated with daily hours of smartphone usage. There is a need for longitudinal studies to find out reasons for the overuse of smartphone devices among Saudi Arabian medical students as well as to examine the link smartphone addiction have to stress and anxiety.

### Author`s Contribution

**AAA and SHA** conceived and designed the study, contributed in data collection, and manuscript drafting.

**MB, EMS and AA** contributed in data collection, statistical analysis, and manuscript writing.
